# Recent advancements in sample pretreatment methods for glycoproteomics

**DOI:** 10.52601/bpr.2025.240072

**Published:** 2026-06-30

**Authors:** Wei Zhang, Siyuan Kong, Weiqian Cao

**Affiliations:** 1 Shanghai Fifth People's Hospital and Institutes of Biomedical Sciences, NHC Key Laboratory of Glycoconjugates Research, Fudan University, Shanghai 200433, China

**Keywords:** Glycoproteomics, Sample pretreatment, Enrichment strategies, High-throughput analysis

## Abstract

Comprehensive glycoprotein analysis is essential for exploring the role of protein glycosylation in diverse biological processes and disease mechanisms. Yet it remains challenging due to the structural complexity and heterogeneity of glycans. Bottom-up glycoproteomics utilizing liquid chromatography-mass spectrometry (LC-MS)-based techniques has emerged as a powerful tool for in-depth protein glycosylation analysis. Sample pretreatment is the first and critical step that significantly influences subsequent chromatographic separation and MS analysis. This review provides an overview of the key steps in current sample pretreatment workflows for glycoproteomics, emphasizing recent advancements in sample preparation and enrichment strategies developed over the past decade. It highlights improvements in enrichment efficiency, compatibility with high-throughput analyses, and applications to biological samples, and also discusses the remaining challenges and future directions for these technologies.

## INTRODUCTION

Glycosylation, as one of the most prevalent post-translational modifications (PTMs) in living organisms, plays a crucial role in protein diversity and biological functions. Among over 200 known PTMs, glycosylation significantly impacts various biological processes, such as immune response, cell signaling, and protein folding (Reily *et al.* 2019; Schjoldager *et al.* 2020; Smith and Bertozzi 2021). Its involvement in diseases like cancer, neurodegenerative disorders, and metabolic conditions underscores the importance of understanding the dynamics and extent of glycosylation across the proteome, which is essential for both basic research and clinical applications (Chakraborty *et al.* 2025; Dai *et al.* 2024; Su *et al.* 2022b). However, the diversity of glycosylation types and their dependence on cellular and disease states make the accurate identification and quantification of glycosylation modifications particularly challenging.

Despite its importance, comprehensive analysis of glycosylation remains challenging due to the complexity and heterogeneity of glycan structures. Recent advancements in bottom-up glycoproteomics strategies, particularly LC-MS-based techniques, have provided effective approaches for in-depth glycoproteome analysis. The workflow typically involves several key steps, including sample pretreatment, LC separation, MS analysis, and data interpretation. Among these, the sample pretreatment step plays a critical role in influencing subsequent chromatographic separation and MS analysis, making it a key determinant of the success of glycoproteomic studies.

The basic pretreatment workflow for glycoproteomics follows that of traditional proteomics, with the addition of specific enrichment steps to capture glycopeptides, which are often present at low abundance in complex samples (Xiong *et al.* 2024). This involves a series of steps, including protein extraction, enzymatic digestion, and glycopeptide enrichment. These steps are essential for overcoming the challenges posed by the low abundance and structural complexity of glycopeptides. In recent years, significant progress has been made in developing advanced pretreatment techniques to isolate and analyze the glycoproteome with high sensitivity and specificity. These innovations are crucial for achieving effective chromatographic separation and MS analysis, thereby enabling a comprehensive understanding of the glycosylation modifications in biological samples.

This review summarizes the key steps in the current sample pretreatment workflows for glycoproteomics, highlighting the latest advancements in sample preparation and enrichment strategies developed over the past decade. We discussed improvements in enrichment efficiency, the compatibility of these methods with high-throughput analysis, and their application in biological samples. Additionally, we provided an outlook on the remaining challenges and the future directions for these technologies.

## SAMPLE PREPARATION

**Figure 1:**
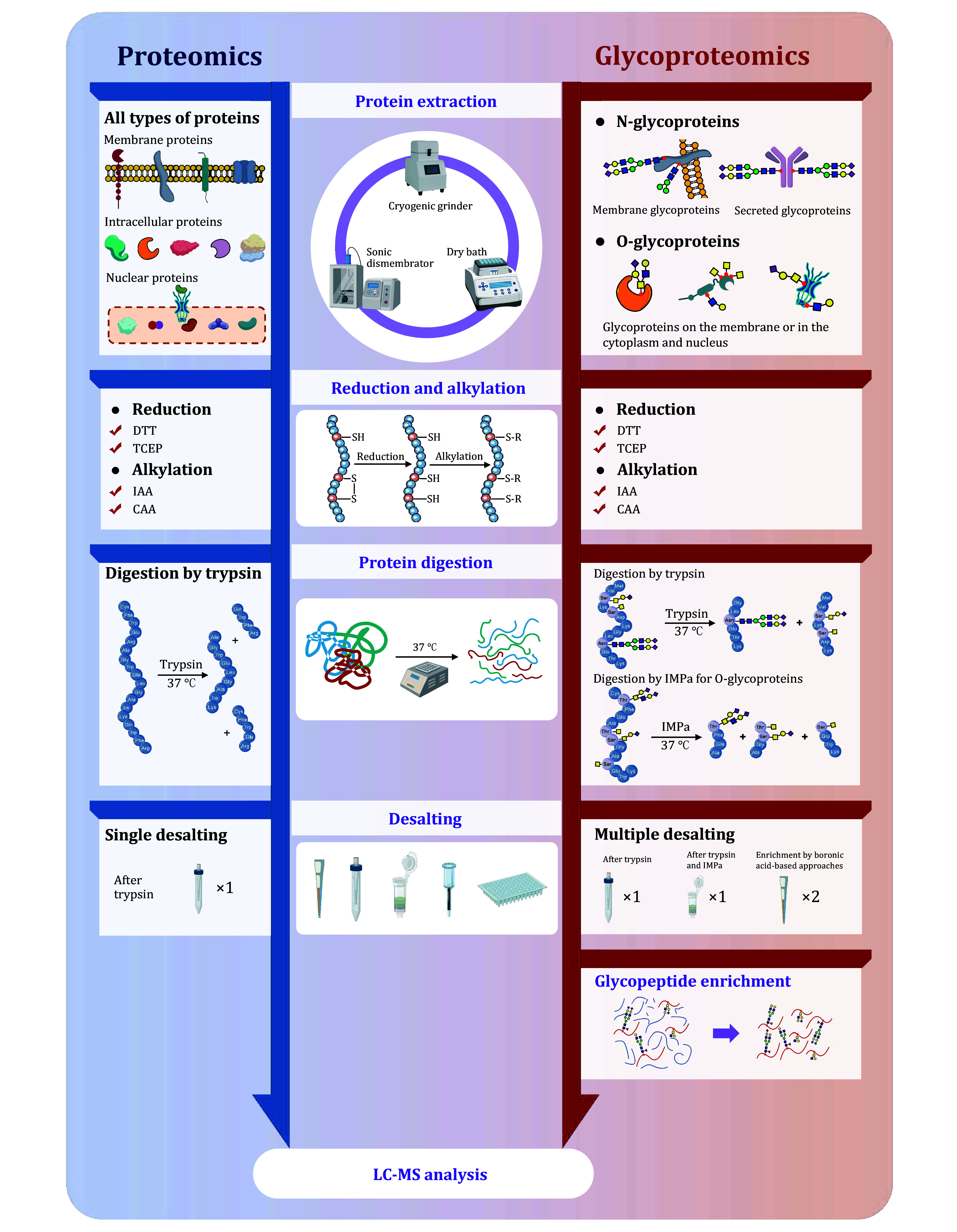
Schematic overview of sample preparation. This figure illustrates the sample preparation workflow for glycoproteomics, along with a comparative overview of sample preparation methods utilized in proteomics. Some of the image elements were derived from the Generic Diagramming Platform (GDP, https://BioGDP.com) (Jiang *et al.* 2025)

The bottom-up proteomic analysis involves extracting protein mixtures from biological samples to uncover the protein composition and dynamics within organisms. Glycoproteomic analysis builds upon this foundation by incorporating an enrichment step (Fig. 1). Sample preparation methods typically include protein extraction, reduction and alkylation, digestion, and desalting. The primary goal of these processes is to comprehensively extract proteins, eliminate impurities such as nucleic acids and lipids, and generate purified peptides to prevent interference with subsequent LC-MS analyses. Consequently, thorough protein extraction and enzymatic digestion constitute the crucial initial steps for downstream applications. This section summarizes the protein extraction, reduction and alkylation, digestion, and desalting methods widely used in glycoproteomics. These procedures vary depending on the sample types to maximize protein extraction efficiency and enhance the quality of subsequent mass spectrometry analyses (Yates *et al.* 2009).

### Protein extraction

Protein extraction methods for tissues, cells, and body fluids in glycoproteomics are tailored to ensure comprehensive protein recovery while considering the localization of different glycosylation types. For instance, mature N-glycoproteins are primarily found in the membrane or secreted proteins (Aebi 2013), whereas O-glycosylation can occur on membrane proteins, in the cytoplasm, or even within the nucleus (Flynn *et al.* 2021; Zhou *et al.* 2022).

For tissue samples, the process involves rinsing the tissues in a frozen buffer to remove blood, rapidly freezing them in liquid nitrogen, and then grinding them using various methods. Manual grinding with pestles and mortars made of glass or polytetrafluoroethylene (PTFE), which have non-stick surfaces, minimizes sample loss. Grinding in liquid nitrogen helps mitigate heat-induced protein degradation, preserving protein integrity (Mertins *et al.* 2018). Tissue samples often present additional challenges due to their diverse cell types, extracellular matrix components, and biomolecular contents. Formalin-fixed paraffin-embedded (FFPE) tissues require special treatment including deparaffinization and crosslink reversal. High-throughput methods, such as adaptive focused acoustics (AFA) technology, can process up to 96 FFPE samples within four hours (Pujari *et al.* 2023).

The samples obtained after tissue grinding, similar to cell samples, require thorough protein extraction, particularly for recovering heavily glycosylated membrane proteins. Disruption of the cell membrane is achieved using a lysis buffer containing surfactants, such as Sodium Dodecyl Sulfate (SDS) (Botelho *et al.* 2010), combined with ultrasonic treatment (Kwon *et al.* 2020). Ultrasonication also breaks down nucleic acids, preventing their aggregation and potential adsorption of proteins, which result in sample loss. To inhibit proteases and phosphatases released during this process and preserve the original protein composition, samples should be maintained at low temperatures, and enzyme inhibitors should be added. Common inhibitors include phenylmethylsulfonyl fluoride (PMSF), 4-[2-aminoethyl]benzenesulfonyl fluoride hydrochloride (AEBSF), ethylenediaminetetraacetic acid (EDTA), ethylene glycol tetraacetic acid (EGTA), and peptide protease inhibitors such as leupeptin, pepstatin, aprotinin, and bestatin (Havanapan and Thongboonkerd 2009; Hooper 2002).

Protein precipitation is a critical step in removing surfactants used during protein extraction, as these surfactants can significantly interfere with subsequent enrichment and mass spectrometry analysis. Organic solvents, such as acetone, often supplemented with sodium deoxycholate, are commonly employed to facilitate protein precipitation. The resulting protein precipitate is collected and then thoroughly washed with precooled solvent to eliminate contaminants, yielding purified proteins (Yun *et al.* 2020). Acetone precipitation is particularly favored due to its efficiency in rapidly dissolving non-polar contaminants, including lipids (Joy *et al.* 2018; Shao *et al.* 2024; Tubaon *et al.* 2017; Zhang *et al.* 2020).

Clinical blood samples, such as plasma and serum, are commonly used in glycoproteomic analysis due to the association of glycoprotein alterations with various diseases. These samples are highly complex, containing approximately 10,000 different proteins, with concentrations ranging from 35–50 mg/mL of albumin to low-abundance proteins at the pg/mL level (Nanjappa *et al.* 2014). The presence of high-abundance proteins poses a significant challenge, as they can mask the detection of low-abundance proteins. Additionally, the heterogeneous nature of glycosylation further complicates the analysis. Blood samples are typically subjected to gentle denaturation methods, such as alternating heating at 100°C and cooling on ice, to prevent the precipitation of high-abundance glycoproteins like IgG, which could interfere with subsequent enzymatic digestion (Eldjarn *et al.* 2023; Hanash *et al.* 2008). To enhance the analytical sensitivity for low-abundance proteins, high-abundance protein depletion is often necessary. The Thermo Abundant Protein Depletion for Mass Spectrometry Kit utilizes immunoaffinity techniques to remove the most abundant proteins, thereby improving the detection of lower-abundance proteins in both discovery and targeted proteomics analyses (Cao *et al.* 2021). Immunodepletion methods, including spin columns, LC, and magnetic beads, can effectively remove up to 20 of the most abundant plasma proteins (Beer *et al.* 2017; Pringels *et al.* 2018; Wu *et al.* 2016). Viode *et al*. introduced a reproducible biochemical plasma depletion method, which utilizes the strong acidity, high ionic strength, and protein denaturation capabilities of perchloric acid to selectively precipitate and subsequently remove high-abundance proteins (Viode *et al.* 2023). A novel methodology has been developed that employs nanoparticle protein coronas to significantly improve the detection sensitivity of low-abundance proteins in serum samples, consequently expanding the depth of proteomic identification coverage (Blume *et al.* 2020).

After protein extraction, all samples must undergo protein concentration quantification. This step is crucial in glycoproteomic sample preparation because the low abundance of glycosylation requires an adequate starting amount of protein to ensure sufficient enrichment for mass spectrometry detection. Approximately 100 µg of starting protein is typically needed for a single mass spectrometry analysis (Bai *et al.* 2018). Protein concentration is measured prior to protein digestion to ensure consistent protein input for subsequent experiments. Quantification methods include the BCA assay, Lowry assay (Smith *et al.* 1985), and Bradford assay (Bradford 1976).

### Reduction and alkylation

Disulfide bonds are covalent linkages commonly found in protein structures, playing a crucial role in stabilizing their tertiary and quaternary configurations. In glycoproteomics, reduction and alkylation are essential steps to disrupt these bonds, enabling the unfolding of protein structures and preparing them for subsequent enzymatic digestion. Reduction involves breaking disulfide bonds using reducing agents, while alkylation modifies the resulting free thiol groups to prevent the reformation of the bonds. These processes not only facilitate effective protein denaturation but also ensure consistency and accessibility for downstream analyses. The choice of reagents and methods for reduction and alkylation must balance efficiency with minimal interference, optimizing conditions for accurate glycoproteomics workflows.

Common disulfide bond-reducing agents include Dithiothreitol (DTT) (Domingos *et al.* 2024), β-mercaptoethanol (Müller and Winter 2017), and Tris (2-carboxyethyl) phosphine (TCEP) (Schlecht *et al.* 2023). DTT is a widely used reducing agent that cleaves disulfide bonds between cysteine residues in proteins. The reduction mechanism involves DTT donating electrons to the sulfur atoms in the disulfide bonds, leading to bond cleavage. β-mercaptoethanol can also reduce disulfide bonds and shares similar reducing properties with DTT. Additionally, β-mercaptoethanol prevents the erroneous formation of disulfide bonds between free cysteine residues, particularly during protein refolding processes. Recently, TCEP, a highly efficient and odorless reducing agent, has gained popularity in glycoproteome research. TCEP exhibits higher stability, does not contain thiol groups that might react with downstream thiol-modifying reagents, and does not require removal before subsequent steps. Moreover, TCEP reacts quickly, making it advantageous for thiol-crosslinking experiments. After disulfide bond reduction, the free-SH groups are typically alkylated using iodoacetamide (IAA). However, due to the potential for multiple side reactions (Boja and Fales 2001), several structural analogs have been developed as alternatives, including iodoacetic acid (IAC), chloroacetamide (CAA), and acrylamide (AA) (Liu and Fitzgerald 2016; Paulech *et al.* 2013).

Proper reduction and alkylation are particularly critical in glycoproteomics, as they ensure complete denaturation of glycoproteins, thereby improving the efficiency of glycopeptide enrichment and enhancing the reliability of mass spectrometry analysis.

### Protein digestion

After alkylation during sample preparation, proteins are digested using proteases to generate peptide fragments suitable for mass spectrometry analysis. Enzymes such as trypsin, trypsin B, Lys-C, Glu-C, and chymotrypsin are commonly used for protein digestion. Trypsin, the most widely used protease (Olsen *et al.* 2004), cleaves proteins at the C-terminal of lysine (Lys) and arginine (Arg) residues with high specificity. This digestion typically generates peptide fragments with charges of +2 or +3, making them ideal for LC-MS analysis due to favorable *m*/*z* ratios (Tyers and Mann 2003). Lys-C cleaves predominantly at the C-terminal of Lys residues, offering an alternative option for highly specific digestion (Wu *et al.* 2018b). Glu-C, another commonly used enzyme, cleaves at the C-terminal of glutamic acid (Glu) and, under certain conditions, aspartic acid (Asp), adding flexibility in tailoring digestion strategies to specific sample requirements (Hansen *et al.* 2018). Chymotrypsin, a serine protease, cleaves at the C-terminal of aromatic amino acids such as tyrosine (Tyr), tryptophan (Trp), and phenylalanine (Phe) (Giansanti *et al.* 2016).

Significant research efforts have been directed toward the structural modification of trypsin to enhance its proteolytic activity and thermal stability. While conventional enzymatic digestion protocols typically employ 37°C as the standard reaction temperature, recent advances in protein engineering have yielded thermostable trypsin variants capable of maintaining catalytic efficiency at elevated temperatures. This thermal tolerance potentially obviates the requirement for conventional reduction and alkylation procedures. The mechanism of enhanced cleavage efficiency at higher temperatures can be attributed to temperature-induced protein denaturation, which disrupts tertiary structure and increases solvent accessibility of disulfide bonds, thereby facilitating their enzymatic hydrolysis.

Notable commercial developments in this field include Merck's recombinant trypsin (rTrypsin), which demonstrates robust stability at 57°C with complete digestion achieved within 1 h. Similarly, Promega's Rapid Digestion Trypsin Kit incorporates an optimized buffer system that enables enzymatic reactions at 70°C, effectively reducing processing time to 1 h. These technological advancements offer particular advantages for glycoproteomic applications, where the elimination of reduction/alkylation steps simplifies experimental workflows, minimizes manual sample manipulation, and consequently reduces risks of sample degradation or exogenous contamination. Nevertheless, for proteins containing complex disulfide bond networks, high temperatures alone may not fully eliminate the need for reduction and alkylation treatments, thereby necessitating a supplemental chemical reduction in specific cases (Ren *et al.* 2021; Wang *et al.* 2011).

In glycoproteomics, combining multiple proteases enables a more comprehensive analysis of glycopeptides and their modifications, providing deeper insights into glycosylation patterns. For instance, certain glycoproteins have glycosylation sites flanked by few enzyme-accessible amino acids, limiting effective cleavage by trypsin alone (Li *et al.* 2022). In such cases, a combination of proteases is utilized to ensure that glycopeptides remain within an optimal size range for mass spectrometry detection, preventing overly large fragments that could complicate analysis. This tailored approach enhances the efficiency and precision of glycopeptide profiling, advancing glycoproteome identification by MS-based analysis (Kang *et al.* 2024; Tkalec *et al.* 2024). In protein regions with dense O-glycosylation, incomplete enzymatic digestion caused by glycan steric hindrance often prevents effective detection of these sites. To address this challenge, the immunomodulating metalloprotease IMPa has been developed to specifically cleave at the N-terminus of O-glycosylated serine or threonine residues (motif: X-S/T-X`, X ≠ I/R/D), functioning across all major O-glycan core structures regardless of the presence of sialic acids (Kang *et al.* 2024). Studies demonstrate that IMPa processes glycopeptides requiring at least two flanking amino acids at the O-glycosylation site (Vainauskas *et al.* 2022). The mucinolytic enzyme OpeRATOR, derived from the human gut symbiont *Akkermansia muciniphila*, selectively hydrolyzes O-glycopeptides at the N-terminal side of glycosylated serine/threonine residues, producing site-specific O-glycopeptide fragments with preserved glycan structures (Yang *et al.* 2018b). Nevertheless, its recognition specificity is suboptimal for sialylated glycan structures (Yang *et al.* 2018a, 2020). Their unique cleavage mechanism enables precise mapping of O-glycosylation sites while maintaining glycan integrity for structural analysis.

### Desalting

Peptide desalting aims to remove residual salts (such as ammonium bicarbonate and phosphates) after protein digestion to minimize their interference with glycopeptide enrichment and LC-MS/MS analysis (Urban 2022). Multiple desalting steps are often necessary to ensure compatibility with downstream applications (Fig. 1). Salts could disrupt glycopeptide enrichment using methods such as zwitterionic hydrophilic interaction chromatography (ZIC-HILIC) or boronic acid-based approaches, which are sensitive to pH and ionic strength. Additionally, salt contamination in MS could interfere with the electrospray ionization (ESI) process, reducing ionization efficiency and potentially clogging the ion source (Nestor *et al.* 2023). Salts can also adversely affect chromatographic separation, data quality, and protein identification. Salt removal leverages differences in molecular size, charge characteristics, and solubility achieved through physical or chemical methods. Standard desalting techniques include dialysis, solid-phase extraction (SPE) (Enders *et al.* 2012), centrifugal filtration, and ultrafiltration. After desalting, samples are typically concentrated or re-dissolved in a mass spectrometry-compatible buffer, ensuring high-quality MS data acquisition.

Dialysis is a traditional desalting technique widely used to remove small molecule salts and other low molecular weight impurities. This method employs a semipermeable membrane that allows small molecules (*e*.*g*., salts and buffer components) to diffuse through while retaining larger molecules (*e*.*g*., peptides and proteins) within the inner chamber. Dialysis is simple, cost-effective, and suitable for small-scale experimental processing. However, careful control of conditions is essential to avoid sample degradation or excessive concentration (Wu *et al.* 2018a). As the mildest desalting method, dialysis effectively removes low concentrations of salts but has drawbacks, such as lengthy processing times and limited suitability for high-throughput applications.

SPE is a widely used desalting method in proteomics and PTM studies. SPE utilizes the affinity of the stationary phase to adsorb and separate salts from peptides. The process involves conditioning the C18 SPE column with organic solvents (*e*.*g*., acetonitrile), followed by equilibration with acidified water (*e*.*g*., 0.1% formic acid or trifluoroacetic acid) (Schmelter *et al.* 2018). Peptides are loaded onto the column, and retained while contaminants are washed away with acidified water. The peptides are then eluted using an organic solution (*e*.*g*., acetonitrile:water (4:1) acidified with 0.1% formic acid), followed by drying (Bladergroen and van der Burgt 2015; Schmelter *et al.* 2018). SPE is known for its high recovery rates and efficiency, making it a preferred choice in glycoproteomics.

Centrifugal filtration and ultrafiltration are rapid and efficient desalting methods that employ membranes with defined pore sizes to separate small molecules, such as salts and buffer components, from larger molecules. By applying centrifugal force or moderate pressure, small molecules pass through the membrane, while larger molecules are retained (Yu *et al.* 2024). This technique is particularly suitable for high-throughput processing of complex samples. Additionally, centrifugal filtration allows for simultaneous desalting and sample concentration, enhancing analytical sensitivity (Nomura *et al.* 2020). However, the potential clogging of the membrane by larger molecules may reduce filtration efficiency, so appropriate sample handling is essential.

## GLYCOPEPTIDE ENRICHMENT

Glycosylation, as one of the most complex PTMs, is often present at sub-stoichiometric levels (Hao *et al.* 2011). This low abundance, coupled with the structural complexity of glycan modifications, makes glycosylation particularly challenging to analyze (Chau *et al.* 2023; Kong *et al.* 2022). Due to its low stoichiometry, glycopeptides can easily be impressed by a large number of non-glycopeptide signals in MS analyses. Therefore, efficient enrichment of glycopeptides is critical to ensure that glycosylation-related data is not lost amidst background signals from non-modified peptides (Gutierrez-Reyes *et al.* 2022; Suttapitugsakul *et al.* 2020).

Enrichment methods for glycosylation typically rely on the specific interactions between the glycan moiety and various binding materials. Based on the principles of these enrichment techniques, we will review recent advancements in enrichment methods, including hydrazide chemistry, lectin-based affinity strategies, boronic acid-based approaches, and hydrophilic interaction liquid chromatography (HILIC). The recently developed enrichment methods and their characteristics are summarized in Table 1.

**Table 1:** Overview of recently developed glycopeptide enrichment methods and their characteristics

Strategies	Methods	Characteristics	Reference
Hydrazide chemistry-based enrichment	CHO-GlcNAc	The O-GlcNAc glycopeptides were labeled with a Gal moiety, and the aldehyde groups were introduced. Then, the labeled O-GlcNAc glycopeptides could be efficiently enriched	Chen *et al.* 2021
	Fe_3_O_4_@PMAH	The abundant hydrazide groups enable highly specific enrichment of glycopeptides and the magnetic core makes it suitable for high-throughput sample processing	Liu *et al.* 2014
	Thermosensitive polymer	Efficient covalent coupling between N-glycoproteins/glycopeptides and polymer chains is facilitated by reduced mass transfer resistance and accessible functional groups. Additionally, the thermosensitive polymer can be easily precipitated and recovered by raising the temperature above 34 °C	Bai *et al.* 2018
Lectin-based enrichment	Multi-lectins	The combination of ConA, Jac, and WGA amplifies the lectin–glycoprotein interaction via the glycoside cluster effect	Lee *et al.* 2012
	Lectin combines FASP	It integrates enzymatic digestion and enrichment by binding to lectins on the top of a filter without the additional lyophilization	Zielinska *et al.* 2010
	BC2L-A	BC2L-A enabled specific enrichment of C-and O-mannosylated peptides	Hütte *et al.* 2022
Boronic acid-based enrichment	Benzoboroxole	By using a boronic acid derivative (benzoboroxole) and conjugating it onto a dendrimer, the boronic acid-sugar interactions were enhanced	Xiao *et al.* 2018
	MASC	Ultrathin C_3_N_4_ nanosheets are hydrophilic and exhibit good stability in both acidic (pH = 3) and alkaline (pH = 11) environments. This overcomes the drawbacks of traditional boronic acid materials, which suffer from degradation of labile glycans in alkaline media	Zhang *et al.* 2019
	GO@mSiO_2_−GLYMO−APB	The combination of a high surface area, size exclusion properties from mesoporous materials, and hydrophilic interactions from silicon materials collectively enhances the specificity for glycopeptides	Kong *et al.* 2021
	GMA and APB	Chitosan has been utilized to create nanosphere support materials with excellent dispersibility, biocompatibility, chemical stability, and improved specificity for glycopeptides	Zou *et al.* 2012
	Fe_3_O_4_@P(AAPBA-co-monomer) NPs	The resulting hydrophilic NPs exhibit an enhanced binding capacity toward glycoproteins by an additional functional monome complementary to the surface presentation of the target protein	Zhang *et al.* 2015
	magG@PF@APB	The composites gathered strong magnetic responsiveness, a large surface area, and excellent biocompatibility	Wang *et al.* 2015
	GMA-MAA-DVB polymer	Material possessed high sensitivity for glycoproteins and glycopeptides (0.04 ng /mL) and (1 fmol/L)	Mujahid Ali *et al.* 2020
HILIC-based enrichment	Fe_3_O_4_@TpBD@Au@GSH	The native hydrophilic TpBD and the highly hydrophilic GSH furnished the composite with dual-hydrophilic performance	Su *et al.* 2022a
	DEAE-Sepharose	It integrated the advantages of Click Maltose and zwitterionic HILIC (ZIC-HILIC) and showed a relatively higher specificity for glycans	Zhu *et al.* 2017
	HBS	Superior hydrophilicity and switchable-charge ensure high sensitive to sialylated glycopeptides	Dong *et al.* 2017
	Acid-assisted de-sialylation method	It combines HILIC enrichment with chemical de-sialylation. Over 90% of sialic acid residues were removed from bovine fetuin using acid-assisted de-sialylation, simplifying the glycan structure and improving identification sensitivity	You *et al.* 2018
	ZICF-PAMAM	Multiple branched structure and good solubility facilitated strong interactions with glycopeptides	Cao *et al.* 2016
	GO-PEI-Au-L-Cys ZIC-HILIC	The high selectivity and reduced enrichment time for glycopeptides are ensured by the large surface area and superior hydrophilicity of these composites	Jiang *et al.* 2014
	GO-Fe_3_O_4_/SiO_2_/AuNWs/L-Cys	Good biocompatibility of GO, strong magnetic responses of Fe_3_O_4_, large surface area of ultrathin Au nanowires for the enrichment of glycopeptides	Jiao *et al.* 2017

### Hydrazide chemistry-based enrichment

Hydrazide chemistry-based enrichment exploits the chemical reactivity of cis-diol groups in glycans. In this method, carbohydrates are oxidized by sodium periodate to generate aldehydes, which then form covalent bonds with hydrazides immobilized on solid supports or magnetic beads (Bladergroen and van der Burgt 2015). The hydrazone reaction specifically targets the reducing ends of glycans on glycoproteins, enabling selective capture and minimizing non-specific binding to other proteins. This selectivity makes the method highly effective for enriching glycoproteins or glycopeptides from complex biological samples.

The high specificity and selectivity of this strategy have led to the development of several enrichment methods. Chen *et al*. developed the CHO-GlcNAc strategy to enrich O-GlcNAc glycopeptides. In this strategy, O-GlcNAc glycopeptides are first labeled with Gal molecules, followed by chemical oxidation to introduce an aldehyde group. These labeled O-GlcNAc glycopeptides can then be enriched via the equilibrium between hydrazone and oxime bonds (Chen *et al.* 2021). Liu *et al*. synthesized a hydrazide-functionalized core-shell magnetic nanocomposite, Fe_3_O_4_@poly (methacrylic hydrazide) (Fe_3_O_4_@PMAH), for selective enrichment of N-glycopeptides. In three repeated experiments, they identified 175 unique glycopeptides and 181 glycosylation sites, corresponding to 63 unique glycoproteins, with an enrichment specificity of 69.6% for glycopeptides and 80.9% for glycoproteins (Liu *et al.* 2014). Bai *et al*. developed an acyl hydrazone-functionalized thermosensitive polymer, which can easily precipitate and be recovered when the system temperature exceeds 34°C. This polymer enriched and identified 329 N-glycosylation sites in plasma exosomes (Bai *et al.* 2018).

However, due to the irreversible nature of glycan release, this approach is primarily used for glycosylation site analysis (Zhang *et al.* 2003). In this process, endoglycosidase such as PNGase F treatment releases glycans by converting Asn to Asp, allowing for the identification of N-glycosylation sites through mass spectrometric analysis of the deglycosylated peptides (Zhang *et al.* 2003). As a result, this method is less suitable for intact glycopeptide analysis (Song *et al.* 2014).

### Lectin-based enrichment

Lectin-based affinity enrichment is another widely used strategy in glycoproteomics, leveraging the specific recognition of distinct glycan structures for the reversible capture of glycopeptides and glycoproteins. Each lectin selectively binds to specific glycan types, making it ideal for both glycosylation site analysis and the enrichment of intact glycopeptides. This reversibility offers a significant advantage in enriching glycoproteins or glycopeptides from complex biological samples, allowing for more comprehensive analyses.

For instance, Lee *et al*. optimized native multi lectin affinity chromatography, combining concanavalin A (ConA), jacalin lectin, and wheat germ agglutinin (WGA) to fractionate glycoproteins from MCF-7 breast cancer cell lysates (Lee *et al.* 2012). These lectins enable the efficiency of capturing glycoproteins based on their glycan contents. Mann *et al*. advanced lectin-based enrichment by developing a "filter-aided sample preparation" (FASP) method, which combines lectins with peptides on the filter surface to enrich glycopeptides. This approach enabled the identification of 6367 N-glycosylation sites on 2352 proteins from mouse tissues and plasma using high-precision mass spectrometry, significantly enhancing the localization of protein N-glycosylation sites (Zielinska *et al.* 2010). Subsequently, they extended the FASP method by incorporating multiple enzymes (MED-FASP), which further improved the comprehensive identification of peptides, proteins, and PTMs (Wiśniewski and Mann 2012). Other lectin-based strategies, such as lectin affinity chromatography (Heo *et al.* 2007) and lectin-functionalized magnetic beads (Choi *et al.* 2011), are also developed, each offering unique advantages for different experimental needs.

For glycosylation types that are challenging to enrich using traditional chemical methods or hydrophilic enrichment materials, lectin-based strategies provide an effective alternative. For example, Hütte *et al*. discovered that the α-mannose-specific *Burkholderia cenocepacia* lectin A (BC2L-A) selectively enriched C- and O-linked mannosylated peptides, offering superior specificity compared to other mannose-binding lectins (Hütte *et al.* 2022).

The increasing diversity and complexity of lectins used in glycoproteomics have led to the creation of specialized databases that catalog lectin specificity, structure, function, and origin. The Glyco3D portal, for example, provides detailed three-dimensional structural data and related lectin information (Sarkar *et al.* 2015). Additionally, the UniLectin3D project integrates glycan-related biological information from multiple sources, consolidating lectin databases, structural biology resources, and glycan modification repositories into a comprehensive platform (Bonnardel *et al.* 2019). These resources enable researchers to select and optimize lectin affinity chromatography conditions with greater precision.

### Boronic acid-based enrichment

Boronic acid-based enrichment methods utilize the affinity of boronic acid for the cis-diol structure present in monosaccharides, enabling efficient enrichment of glycopeptides (Lee *et al.* 2005). This strategy works by forming reversible covalent bonds between boronic acid and the diol groups in glycopeptides, which enhances the recovery efficiency of glycopeptides from complex biological samples (Ahn *et al.* 2015). The captured glycopeptides or glycoproteins can be eluted by using buffers containing high concentrations of monosaccharides or by adjusting the pH to a lower level (Zhang *et al.* 2014). Due to its low cost and compatibility with mass spectrometry, boronic acid-based capture has become a widely used technique for enriching glycopeptides. However, conventional boronic acid affinity materials exhibit relatively low affinity for cis-diol glycan chains, leading to suboptimal capture efficiency and selectivity, particularly in samples with high glycan density or complex glycosylation patterns (Xiao *et al.* 2018).

To address these issues, recent studies have focused on improving the performance of boronic acid affinity materials. For example, Wu *et al*. enhanced the interaction between boronic acid and glycans by using a boronic acid derivative, benzoboroxole, which was conjugated to a dendritic polymer. This synergistic approach enabled more effective glycosylation analysis by providing site-specific and glycan structural information (Xiao *et al.* 2018). Ying *et al*. developed a novel, self-assembling boronic acid-functionalized and Au-doped straticulate C3N4 (MASC), which showed enhanced affinity for glycopeptides. Using this method, they identified an average of 1465 glycopeptides from 839 glycoproteins in female urine samples and 1553 glycopeptides from 884 glycoproteins in male urine samples, all from a single mass spectrometry analysis (Zhang *et al.* 2019).

Further innovations in boronic acid capture technologies have led to new enrichment strategies. Kong *et al*. proposed a boronic acid-functionalized mesoporous graphene–silica composite (GO@mSiO_2_−GLYMO−APB) for isolating intact glycopeptides from complex biological samples. This composite material combines a high surface area, size exclusion properties from mesoporous materials, hydrophilic interactions from silicon materials, and the synergistic effects of reversible covalent bonding with boronic acid. Analysis of human serum samples demonstrated the robustness of this strategy in comprehensive glycopeptide analysis (Kong *et al.* 2021). Zou *et al*. use chitosan to create nanosphere support materials modified with glycidyl methacrylate (GMA) and 3-amino phenylboronic acid (APB), enabling selective enrichment of glycopeptides. Their method showed high specificity for glycopeptides, even in complex mixtures such as horseradish peroxidase and bovine serum albumin digestion products (Zou *et al.* 2012).

Other notable advancements include Zhang *et al*.'s development of a time-efficient method using distillation precipitation polymerization (DPP) to synthesize phenylboronic acid and copolymer multifunctional magnetic nanoparticles (NPs). These nanoparticles enhanced glycoprotein binding by incorporating additional functional monomers complementary to the target proteins' surfaces. The resulting hydrophilic Fe_3_O_4_@P(AAPBA-co-monomer) NPs demonstrated strong magnetic responsiveness and excellent glycoprotein separation performance, allowing for easy isolation under an external magnetic field (Zhang *et al.* 2015). Wang *et al*. introduced a straightforward synthesis method for a novel boronic acid-functionalized magnetic graphene@phenolic resin multilayer (magG@PF@APB) composite material. This composite exhibited strong magnetic responsiveness, a large surface area, excellent biocompatibility, and enhanced affinity for boronic acid, making it highly suitable for glycoproteome analysis in complex biological samples, including low-volume human serum (Wang *et al.* 2015). Additionally, Du *et al*. reported a selective method for extracting nucleosides, glycopeptides, and glycoproteins using a boronic acid-functionalized GMA-MAA-DVB polymer. This material possesses sensitivity for glycoproteins and glycopeptides, with detection limits of 0.04 ng/mL and 1 fmol/L, respectively. However, its performance in complex biological samples is suboptimal, as demonstrated by the identification of only six glycoproteins in saliva samples (Mujahid Ali *et al.* 2020).

In conclusion, while boronic acid-based capture technologies have some limitations (Xiao *et al.* 2018), ongoing advancements in material design and functionalization continue to enhance their specificity and efficiency, making them valuable tools for glycoproteomics research.

### Hydrophilic interaction liquid chromatography

HILIC is an indispensable strategy for glycoproteome enrichment (Riley *et al.* 2021). The use of HILIC for glycopeptide enrichment is well-established. Several reviews have provided detailed insights into the HILIC materials, including their advantages and limitations (Brandi *et al.* 2022; Chao *et al.* 2024; Jandera 2011; Jandera and Janás 2017; Sun *et al.* 2019). HILIC was first introduced by Alpert AJ in 1990 as a variation of normal-phase liquid chromatography (Alpert 1990). The technique enriches glycopeptides through the hydrophilic nature of their sugar moieties, although non-glycosylated hydrophilic peptides may also be co-enriched (Mysling *et al.* 2010). HILIC employs a semi-aqueous mobile phase that forms a "water-enriched" layer within the hydrophilic stationary phase. During the separation and enrichment process, hydrophilic glycopeptides migrate from the organic buffer into this water-rich layer (Guo 2015; Hemström and Irgum 2006; van der Plas *et al.* 2023). Further studies have made modifications to HILIC materials to enhance their glycopeptide enrichment capacity, with a particular focus on increasing the density of hydrophilic groups on the stationary phase to improve detection sensitivity and loading capacity (Wang *et al.* 2017).

For example, Su *et al*. synthesized glutathione (GSH)-modified magnetic covalent organic framework (TpBD) composites (Fe_3_O_4_@TpBD@Au@GSH) using a two-step synthesis and post-modification strategy. This approach successfully detected 492 and 160 glycopeptides from 5 µL of human serum and saliva samples, corresponding to 134 and 64 glycoproteins, respectively (Su *et al.* 2022a). Zhu *et al*. employed Diethylaminoethyl sepharose (DEAE-Sepharose) solid-phase extraction microcolumns to enrich N-glycopeptides. Combining the advantages of the click maltose method and the oligomeric ZIC-HILIC method, this strategy provided higher specificity for N-glycopeptides. Subsequent LC-MS/MS analysis identified 219 N-glycosylation sites in 115 N-glycoproteins in serum (Zhu *et al.* 2017). Dong *et al*. developed a hydrophilic interaction and surface-charge-switchable histidine-bonded silica (HBS) material, successfully enriching human serum and capturing 487 glycosylation sites with a selectivity of 92% (Dong *et al.* 2017). Additionally, You *et al*. introduced a glycan simplification strategy that combines HILIC enrichment with chemical deglycosylation to characterize O-GalNAc glycosylation in human serum. This method led to the identification of 185 O-GalNAc-modified peptides from human serum (You *et al.* 2018).

ZIC molecules, containing both cationic and anionic groups within the same molecule, possess an overall neutral charge. This structural feature gives ZIC molecules a strong affinity for water, resulting in the formation of a highly hydrated interfacial layer on the ZIC surface through electrostatic interactions. These interactions enhance retention time on charged stationary phases in HILIC, offering higher selectivity compared to neutral HILIC matrices (Fang *et al.* 2016). Cao *et al*. synthesized a polymeric oligomer (ZICF) based on poly(amidoamine) dendrimers (PAMAM) to enrich glycopeptides. The excellent solubility and branched polysaccharides of ZICF facilitated strong interactions with glycopeptides, achieving an impressive 90% recovery rate with only 0.1 μL of human serum (Cao *et al.* 2016). Jiang *et al*. developed a novel ZIC-HILIC material by loading gold nanoparticles onto the surface of graphene oxide (GO) and immobilizing L-cysteine via gold-sulfur bonds using polyethyleneimine as a reducing and stabilizing agent. This composite demonstrated high selectivity for glycopeptide enrichment from biological samples (Jiang *et al.* 2014). Similarly, Jiao *et al*. synthesized an ultra-thin gold nanowire-assisted oligomeric hydrophilic magnetic graphene oxide (GO-Fe_3_O_4_/SiO_2_/AuNWs/L-Cys) material in four simple and rapid steps. In three replicate analyses of 40 µg of mouse liver proteins, they identified 793 glycopeptides from 467 glycoproteins (Jiao *et al.* 2017).

HILIC, particularly ZIC-HILIC, has become increasingly popular for glycopeptide enrichment due to its simple operation, the fact that it does not introduce salts into the system, and its high compatibility with mass spectrometry. Furthermore, its broad enrichment capability makes it particularly suitable for enriching intact glycopeptides, contributing to its widespread use in glycoproteomics.

## HIGH-THROUGHPUT SAMPLE PREPARATION

**Figure 2:**
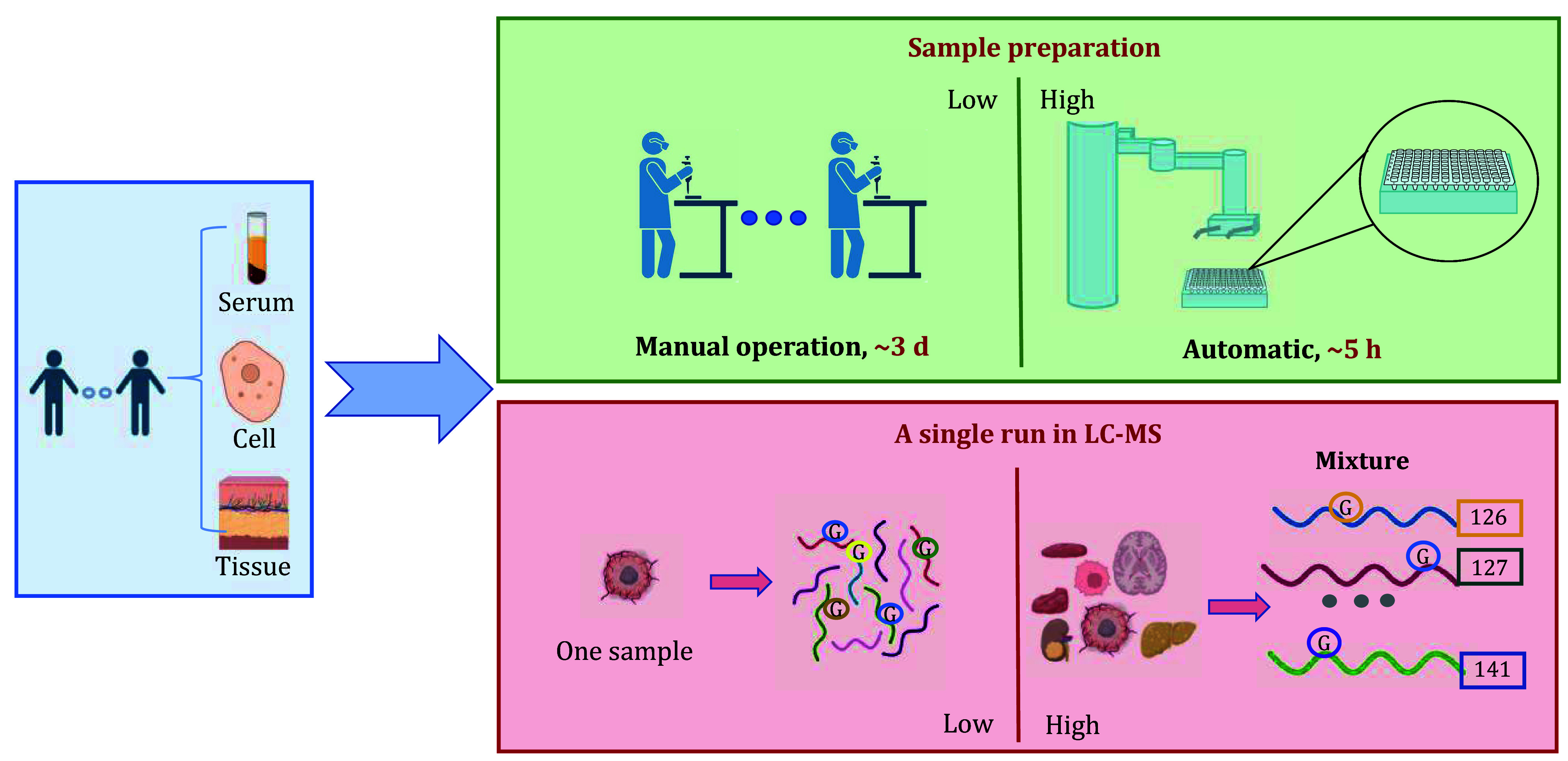
Strategies for high-throughput sample processing. Currently, high-throughput glycoproteomic analysis mainly focuses on high-throughput sample preparation (upper-right part) and new methods for mass spectrometry analysis, such as the use of chemical isotope labeling to enable the identification of different samples in a single MS run (lower-right part). Part of the image elements were created in BioRender (https://www.biorender.com/)

As with proteomics, the demand for large-scale glycoproteomic analyses in clinical research continues to grow. Achieving rapid, high-throughput glycoproteomic analysis while maintaining optimal coverage and depth has become a critical challenge. Sample preparation for mass spectrometry-based glycoproteomics involves numerous tedious and time-consuming steps. Methods relying on manual and independent processing of many samples can lead to material loss, contamination, or errors that significantly affect reproducibility and results (Zhang *et al.* 2013). Moreover, due to the inherent complexity and diversity of glycoproteins, sample preparation often suffers from low reproducibility and throughput in glycoproteomics studies (Wang *et al.* 2024). Automation technology can reduce human errors, improve experimental reproducibility and consistency, and speed up sample processing. Although high-throughput sample preparation methods have been well-developed in proteomics, their application in glycoproteomics remains relatively underdeveloped. Most research in glycoproteomics focuses on improving chromatographic or mass spectrometry methods for enhanced glycopeptide identification (Fig. 2).

For example, Bi *et al*. compared capillary columns of different diameters, lengths, and elution times, leading to the identification of 5218 unique N-glycopeptides in liver cancer tissues and adjacent non-cancerous tissues (Bi and Tian 2024). Sun *et al*. utilized chemical labeling-assisted complementary dissociation to identify and quantitatively analyze intact N-glycopeptides, providing the most comprehensive site-specific and subclass N-glycosylation analysis of human serum immunoglobulin G (IgG) to date (Sun *et al.* 2023). Chen *et al*. introduced a novel tandem S-Trap-IMAC-HILIC strategy called TIMAHAC, which enables high-throughput, automated sequential enrichment of both glycopeptides and phosphopeptides (Chen *et al.* 2024).

As in proteomics, efficient sample preparation methods in glycoproteomics are crucial for reducing analytical biases, improving reproducibility, and enhancing data accuracy, all of which are vital for high-throughput analyses. Several high-throughput sample preparation methods, developed primarily for proteomics, have already shown promise in glycoproteomics. For instance, FASP involves protein digestion on a molecular weight cutoff (MWCO) membrane, enabling rapid clearance of saline samples (Wiśniewski *et al.* 2009). As mentioned in the enrichment section, Mann and colleagues developed and applied lectin-based enrichment techniques based on FASP for glycoproteomic analysis, achieving unprecedented depth of glycosylation site identification. Recently, Yu *et al*. introduced a 96-well filter plate-based FASP method, called 96FASP, for soluble urine concentrates containing approximately 10 μg of total protein, which identifies 700–900 proteins with a 1% false discovery rate (FDR) (Yu *et al.* 2014). The MStern method, developed by Leutert *et al*., addresses the slow infusion rates in FASP by using large-pore polyvinylidene fluoride membranes that allow rapid liquid passage, thus improving throughput in glycoproteomic studies and making it compatible with liquid-handling robots (Berger *et al.* 2015).

The in-StageTip (iST) method performs all sample preparation steps within a single pipette tip, including lysis, reduction, alkylation, digestion, and fractionation, minimizing contamination and sample loss (Kulak *et al.* 2014). When integrated with 96-well iST plates and fully automated robots, this approach significantly boosts throughput, enabling quantitative analysis of hundreds of plasma proteomes from a single 1 μL fingertip sample in just a 20-min gradient (Geyer *et al.* 2016). The Pipette-tip-based Proteomics Technology (SISPROT) integrates protein pre-concentration, peptide extraction, and high-pH reverse-phase peptide fractionation within a single pipette tip, ensuring protein retention and high reproducibility (Chen *et al.* 2016; Lin *et al.* 2018). Tian *et al*. proposed a high-throughput drug target discovery workflow that integrates single-temperature thermal proteome profiling (TPP), automated proteomics sample preparation (autoSISPROT), and data-independent acquisition (DIA) quantification. This workflow processes 96 samples within 2.5 h, significantly enhancing throughput compared to traditional methods (Wu *et al.* 2024). Bead-based separation technologies, such as single-pot solid-phase enhanced sample preparation (SP3)**,** use paramagnetic beads and are especially suitable for small sample amounts. Leutert *et al*. developed an automated high-throughput SP3 method compatible with 96-well plates and introduced Rapid Robotic Phosphoproteomics (R2-P2), utilizing magnetic particles to process protein extracts and generate mass spectrometry-ready phosphopeptides. This method has been used to study the signaling dynamics of the MAPK pathway in yeast (Leutert *et al.* 2019). Müller *et al*. implemented automated SP3 (autoSP3) for tissue lysate processing in a 96-well format, enabling the reproducible quantification of 500–1000 proteins from 100 to 1000 cells (Müller *et al.* 2020). Pujari *et al*. introduced a rapid workflow for FFPE-based proteomics sample preparation using Adaptive Focused Acoustics (AFA) technology. This workflow significantly reduces the overall processing time and is also suitable for small amounts of clinical samples (Pujari *et al.* 2023). Burns *et al*. developed a highly automated 384-well plate sample preparation platform, completing the process from cultured cells to cleaned peptides in about 300 min. This platform can identify and quantify 4000 proteins per sample in a single LC-MS/MS run, using as few as 100 to 10,000 cells (Burns *et al.* 2021).

Most of these high-throughput methods were originally developed for proteomics and have not yet been fully applied in glycoproteomics. However, due to their efficiency in proteomic analyses, these methods hold great potential for advancing glycoproteomic workflows as well.

In glycoproteomics, conventional sample preparation workflows typically require approximately 2–3 d to complete. When combined with the three-hour LC-MS/MS analysis per sample (Kong *et al.* 2022), this protracted processing timeline creates significant constraints on research throughput and operational efficiency. To mitigate these limitations, investigators have developed accelerated digestion strategies including thermal-assisted enzymatic digestion protocols (Shen *et al.* 2021) and ultrasonication-enhanced proteolysis (Huang *et al.* 2023), complemented by streamlined workflow. These innovative methods not only enhance experimental efficiency but also minimize sample loss and potential technical errors, offering promising approaches for high-throughput glycoproteomics applications.

Sample preparation and LC-MS analysis constitute indispensable core components of the entire workflow in glycoproteomic analysis. In high-throughput glycoproteomics research, the efficiency and reproducibility of sample preparation, coupled with the speed and data quality of LC-MS/MS analysis, together represent critical bottlenecks for overall throughput. In small-scale studies, sample preprocessing typically emerges as the critical constraint due to its multi-step nature requiring hours to days for completion, whereas instrument runtime remains relatively predictable. Conversely, in large-scale studies, when sample numbers exceed instrumental capacity, the cumulative LC-MS/MS analysis duration becomes the primary limitation factor.

Minimizing sample volume has emerged as a critical focus in current technological advancements, aiming to reduce the consumption of precious samples while facilitating high-throughput analysis. Previous studies have demonstrated successful glycopeptide enrichment and digestion in 96-well plates with volumes as low as 50 μL (Chen *et al.* 2020; Jiang *et al.* 2019; Momčilović *et al.* 2020; Pucić *et al.* 2011). However, the application of these techniques in 384-well plate systems, which involve sub-20-μL liquid handling, presents several technical challenges that require systematic solutions. First, experimental operation demands the implementation of a high-precision automated platform to ensure accurate dispensing of microliter-scale buffers and reagents. Material selection also plays a critical role, where plates with low adsorption surfaces and advanced sealing mechanisms are imperative to minimize sample loss and evaporation during processing. Second, protocol optimization requires special attention. Both enzymatic digestion and glycopeptide enrichment procedures need to be streamlined through workflow refinement, particularly considering the inherently low abundance of glycosylation modifications. This necessitates the development of more efficient enrichment strategies to improve target recovery rates. Third, analytical sensitivity constitutes a fundamental requirement. The successful detection of low-abundance glycosylation variants ultimately relies on high-resolution mass spectrometry systems capable of maintaining detection reliability at trace concentration levels. In summary, comprehensive methodological advancements spanning from pretreatment workflows to mass spectrometry instrumentation and analytical methods will ultimately enhance the overall sensitivity.

## PROSPECT

The efficiency of each step in glycoproteomic sample preparation significantly impacts the sensitivity of MS analysis. With the continuous advancement of MS technology, especially high-resolution instruments offering exceptional sensitivity and resolution, the detection of low-abundance glycoproteins has become increasingly reliable and precise (Yang *et al.* 2017). As MS instruments continue to improve, future glycoproteomic research will allow for the construction of more comprehensive glycan maps, particularly in complex biological samples. This will enable the identification of a broader range of glycosylation modifications across numerous glycoproteins. More efficient sample pretreatment methods, including high-throughput processing, minimal sample volume handling, and the development of sequential enrichment techniques compatible with other methods, will further enhance the analysis of glycoproteins using these highly sensitive instruments.

As the demand for large-scale, high-throughput glycoproteomic analysis grows, future methods will need to be optimized for processing large numbers of samples efficiently. Automation, advanced sample preparation strategies, and seamless integration with high-throughput MS platforms will be crucial to enable large-scale analyses while maintaining high accuracy and sensitivity. The development of systems capable of handling hundreds or even thousands of samples in parallel will significantly accelerate the discovery of disease biomarkers and provide deeper insights into the role of glycosylation in complex biological contexts.

The ability to analyze minimal sample volumes is particularly important for studying rare cell populations, limited clinical samples, and even single-cell glycoproteomics. As glycosylation is often present at low stoichiometric levels, it is essential to develop more sensitive enrichment techniques that can efficiently capture glycopeptides from even the smallest quantities of biological material. This is especially critical in clinical settings where sample availability is limited, such as in biopsies or circulating tumor cells, enabling more precise approaches to medical research and diagnostics.

To enhance the efficiency of glycopeptide enrichment, future methods should focus on developing integrated approaches capable of efficiently capturing multiple types of glycosylation, such as both N-glycosylation and O-glycosylation, from the same sample. This would enable a more comprehensive analysis of different glycosylation modifications in a single experiment. Furthermore, as glycosylation is known to interact with various other PTMs, such as phosphorylation (Hu *et al.* 2024; Lei *et al.* 2024; Song *et al.* 2019), it is crucial to develop methods that facilitate seamless integration with other PTMs. This will aid in investigating the relationships and potential crosstalk between glycosylation and other PTMs, offering deeper insights into the glycosylation process and its role in cellular functions.

Finally, although the individual steps of sample preparation may seem trivial or inconsequential, each one has a significant impact on the subsequent results. Therefore, developing multi-parameter, controllable, and intelligent analysis systems for quality control (QC) at each stage, ultimately achieving an SOP-level workflow for sample preparation, will be essential. This approach could also represent a promising future application of AI in sample preparation for glycoproteomics.
